# Evaluation of biomarker canine-prostate specific arginine esterase (CPSE) for the diagnosis of benign prostatic hyperplasia

**DOI:** 10.1186/s12917-017-0996-5

**Published:** 2017-03-23

**Authors:** Dora Pinheiro, João Machado, Carlos Viegas, Cláudia Baptista, Estela Bastos, Joana Magalhães, Maria A. Pires, Luís Cardoso, Ana Martins-Bessa

**Affiliations:** 1 University of Trás-os-Montes e Alto-Douro (UTAD), Vila Real, Portugal; 2 Veterinary Teaching Hospital, UTAD, Vila Real, Portugal; 3 Department of Veterinary Sciences, School of Agrarian and Veterinary Sciences, UTAD, Vila Real, Portugal; 4 Centre for the Research and Technology of Agro-Environmental and Biological Sciences (CITAB), UTAD, Vila Real, Portugal; 5 Department of Veterinary Clinics, UPVet, Instituto de Ciências Biomédicas Abel Salazar, Universidade do Porto, Oporto, Portugal; 6 Department of Genetics and Biotechnology, School of Life and Environment Sciences, UTAD, Vila Real, Portugal; 7 UCIBIO, REQUIMTE, Department of Chemical Sciences, Faculdade de Farmácia, Universidade do Porto, Oporto, Portugal; 8 Animal and Veterinary Research Centre (CECAV), UTAD, Vila Real, Portugal

**Keywords:** Prostate, Benign prostate hyperplasia, BPH, Canine prostate-specific arginine esterase, CPSE, Biomarker

## Abstract

**Background:**

Benign prostatic hyperplasia (BPH) is the most common canine prostatic disorder. Although most or even all intact male dogs may develop BPH by 5–8 years of age, many show no clinical signs. Taking into account the non-specific character of clinical and ultrasonographic findings, a new diagnostic approach has recently been proposed based on the augmentation of blood canine prostate-specific arginine esterase (CPSE) in hyperplasic dogs. The aim of the present study was to verify CPSE levels in negative controls and hyperplasic dogs, considering cytological findings as the reference method and taking into account the fact that controls were middle-aged intact dogs (median of 5.0 years), contrarily to previous studies carried out with very young control dogs.

**Results:**

Significant differences of median CPSE levels were found between controls and hyperplasic dogs (29.1 versus 160.7 ng/mL, respectively); and significant positive correlations were found between median CPSE levels and age or prostatic volume (*r* = 0.549 and 0.448, respectively; *p* < 0.001). Sensitivity, specificity, positive and negative likelihood ratios put into evidence the good performance of the test. The agreement between methods was found to be very high, notably between CPSE levels and cytological results (Cohen’s kappa coefficients above 0.8).

**Conclusions:**

Considering the results all together, measurement of CPSE is confirmed as a useful and accurate method and should be considered as an alternative or complementary tool to conventional methods for the diagnosis of BPH in middle-aged dogs.

**Electronic supplementary material:**

The online version of this article (doi:10.1186/s12917-017-0996-5) contains supplementary material, which is available to authorized users.

## Background

Diseases of the prostate are common in middle-aged to older intact dogs. The canine prostate can be affected by benign prostatic hyperplasia (BPH), squamous metaplasia, cysts, inflammation and neoplasia. BPH, the most common prostatic disorder, is a spontaneous disease of intact male dogs that begins as glandular hyperplasia as early as 2 years of age [1]. Dihydrotestosterone is accepted as a key hormone in the pathogenesis of the disease, stimulating enlargement of the prostate by enhancing growth of both the stromal and glandular components [2]. Oestrogens, in particular the ratio of 17β-oestradiol/testosterone, are also involved in the development of BPH, as is potentially prolactin [3] and also other mitogenic growth factors [4, 5].

When present, clinical signs of BPH include serosanguinous urethral discharge not associated with urination [6], hemospermia and hematuria, tenesmus, poor semen quality and infertility, and prostatomegaly [7], as well as constipation and dysuria [3]. The diagnosis of BPH is usually based on history, physical and/or andrologic exam and abdominal ultrasonography; and seldom on cytology by fine needle-aspiration (FNA). Palpation usually reveals a symmetrically enlarged and non-painful prostate. The presence of prostatomegaly confirmed by ultrasonography (with a heterogeneous or hyperechoic parenchyma with or without fluid-filled cysts) is frequently sufficient to reach a conclusive diagnosis of BPH [7]. However, BPH can be difficult to differentiate from other prostatic diseases, including squamous metaplasia, prostatitis, prostatic cysts and abscesses, and prostatic neoplasia, due to the similarity of clinical and ultrasonographic findings. Under these circumstances, other methods should be used, i.e., cytology or biopsy, and more recently the seric quantification of canine prostate-specific arginine esterase (CPSE) [8].

CPSE, a serine protease similar to the human prostate-specific antigen (“PSA”), is the most important specific androgen-dependent protein of prostatic secretion in dog and a remarkable marker of androgenic stimulation [9, 10]. Serum CPSE concentrations, as determined by immunoprecipitation, were found to be significantly elevated in dogs with BPH compared to normal dogs [11]. CPSE determination by enzyme-linked immunosorbent assay (ELISA) is currently an interesting method for BPH diagnosis, especially indicated to early detect dogs with a subclinical pathological condition, and opens new perspectives to the follow-up of medical treatment [8]. However, there is scarce information on CPSE levels in middle-aged healthy dogs. In such animals, the determination of CPSE concentration is a feasible procedure that can be of high importance for early BPH diagnosis.

The aims of the present study were: a) to compare CPSE levels in dogs with BPH (study group) and without BPH (control group); and b) to compare clinical, ultrasonographic and CPSE performances in the diagnosis of BPH, considering the cytological marks as the reference method for this study.

## Methods

### Clinical, ultrasonographic and cytological evaluations

All the dogs (*n* = 60) were evaluated only after an informed consent obtained from their owners. Each dog underwent clinical examination including a thorough history, physical and digital rectal examination (DRE) of the prostate, ultrasonography and cytological evaluation. Clinical manifestations compatible with BPH or cystic prostatic hyperplasia (CPH) comprised constipation, ribbon-like stools, hemorrhagic urethral discharge and/or intermittent hematuria, weight loss, rear limbs pain and abnormal DRE, such as palpable prostatomegaly and painful or abnormal prostate such as nodularity, indurations or asymmetry [12]. Prostatitis was suspected when clinical signs were more severe than BPH, as caudal abdominal or pelvic pain, and a diffuse to asymmetrical enlargement and/or prostatic pain was noted at DRE [13].

**Fig. 1:**
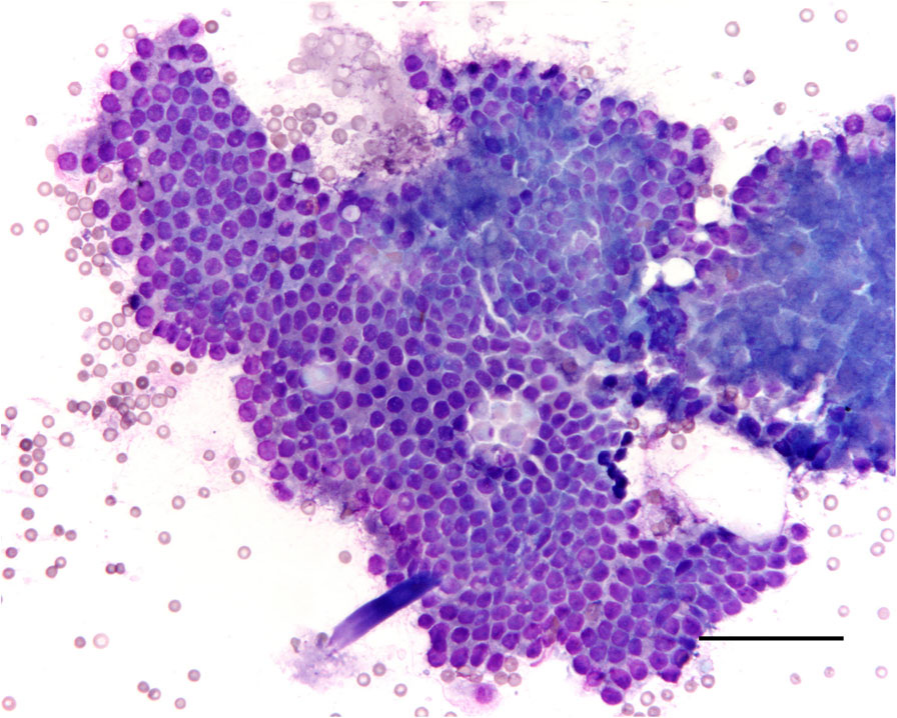
Fine needle-aspirate from a prostate with benign hyperplasia (group A dog). Cells are arranged in clusters and show a uniform morphology with round nucleus and basophilic cytoplasm (Diff-quick staining; bar = 500 μm)

**Fig. 2:**
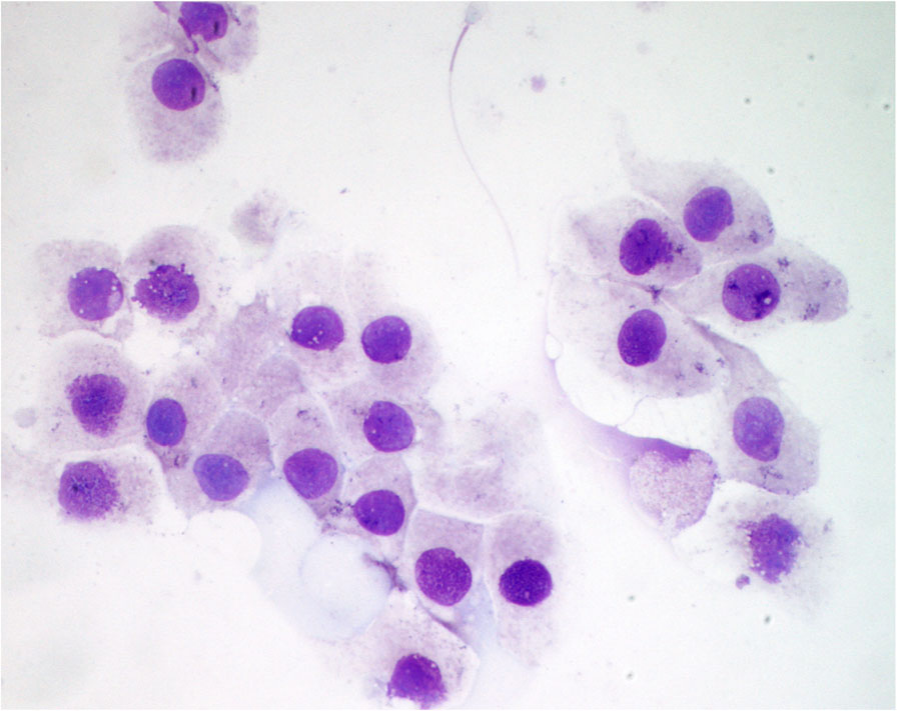
Fine needle-aspirate from a normal prostate (group C dog). Cells are arranged in small clusters with a uniform morphology, showing round nucleus and clear cytoplasm (Diff-quick staining; ×600 magnification)

Additional procedures carried out comprised prostatic ultrasonography, prostatic massage or prostatic FNA for cytological evaluation (Figs. 1 and 2). Transabdominal ultrasonography of the prostate was performed with a Philips HD3 Ultrasound system (Philips Healthcare, USA), using a sectorial 3.0-7.0 MHz and linear 5.0-9.0 MHz transducers. The ultrasonographic examination enabled the evaluation of the prostate size, namely length (L) and height in the longitudinal plan (HL), and height (HT) and width (W) in the transverse one, plus shape, position, internal echogenicity and margins. The values for L, HL/HT and W were the average of three consecutive measurements. Prostatic volume (PV) was determined by the formula PV (cm^3^) = 0.487 × L × W × (HL × HT/2) + 6.38; and the expected volume (EV) was determined by the formula EV (cm^3^) = 8.48 + [0.238 × body weight (kg)] [14]. All variations of the normal ultrasonographic aspect were recorded (i.e., asymmetric shape, size, and aspect of focal or multifocal variations of echogenicity; size and echogenicity of retention cysts or areas of cavitation; and irregular margins). Abnormal records of ultrasonography included BPH-suggestive, cystic prostatic hyperplasia (CPH)-suggestive and prostatitis-suggestive ultrasonograms. Ultrasonographic findings were subdivided into simple BPH, CPH and prostatitis. Findings compatible with BPH included symmetrical prostatic enlargement and diffusely hyperechoic parenchyma, whereas CPH showed hyperechoic parenchyma background with cavitary lesions of varying architecture and distal acoustic enhancement. Prostatitis was suspected when ultrasonographic findings were more pronounced than BPH, being the prostate variably hyperechoic with a more complex contour [13].

For cytology, samples were collected by ultrasound-guided transabdominal FNA, as previously described [6] or by prostatic massage, namely when ultrasonography suggested prostatitis [15]. The cells were then smeared on a slide, air-dried, stained with Diff-Quick and mounted with Entellan^TM^ (Merck Millipore, USA). The presence of uniform sheets or clusters of prostatic cells with middle anysokaryosis and increased size with round and small nucleus, with a basophilic, moderate cytoplasm are the cytological criteria to determine the presence of BPH [16]. These clusters could be in a high quantity but their cells appeared as normal epithelial prostatic ones. The cytology of prostatitis is determined by the presence of a large number of neutrophils associated with a variable numbers of macrophages (more common in chronic prostatitis). Bacteria could be present, both freely or intracytoplasmatically (i.e., inside neutrophils and macrophages). Finally, squamous metaplasia is determined by the presence of prostatic cells with squamous epithelial characteristics such as large and clear to basophilic cytoplasm with small dense nucleus [16].

### Groups of animals

Based on cytological results, two groups of adult intact male dogs were formed, i.e., a study group (A; Table 1; Additional file 1) and a control group (C; Table 2; Additional file 2).

**Table 1:** Records from clinical examination, ultrasonography and cytology in 29 dogs (group A) diagnosed with benign prostatic hyperplasia (BPH) by cytological examination of prostatic aspirates

Clinical examination		Ultrasonography-suggested condition		Cytology (BPH+)	Dogs (n)
Physical signs	DRE	BPH	CPH	Prostatitis	
Absent	Normal	No	No	Yes	1
	Abnormal	No	Yes	No	2
				Yes	1
		Yes	No	No	3
Present	Normal	No	Yes	No	3
				Yes	2
		Yes	No	No	1
	Abnormal	Yes	No	No	5
		No	Yes	No	7
				Yes	4

*CPH* cystic prostatic hyperplasia, *DRE* digital rectal examination

**Table 2:** Records from clinical examination, ultrasonography and cytology in 31 dogs (group C) without benign prostatic hyperplasia (BPH) as determined by cytological examination of prostatic aspirates

Clinical examination		Ultrasonography-suggested condition			Cytology (BPH^−^)		Dogs (n)
Physical signs	DRE	BPH	CPH	Prostatitis	Prostatitis	Metaplasia	
Absent	Normal	No	No	No	No	No	23
					Yes	No	2
	Abnormal	No	Yes	No	Yes	No	1
Present	Normal	No	No	No	No	No	1
				Yes	Yes	No	1
	Abnormal	No	Yes	No	No	Yes	1
					Yes	Yes	1
		Yes	No	No	Yes	No	1

*CPH* cystic prostatic hyperplasia, *DRE* digital rectal examination

Group A included eight mongrels and 21 other dogs from 13 defined breeds, i.e., Labrador Retriever (*n* = 4), Boxer (*n* = 3), Estrela Mountain Dog (*n* = 3), Golden Retriever (*n* = 2), Beagle (*n* = 1), Brittany (*n* = 1), Chow-Chow (*n* = 1), Cocker Spaniel (*n* = 1), Dalmatian (*n* = 1), Pekingese (*n* = 1), Portuguese Sheepdog (*n* = 1), Rottweiler (*n* = 1) and Siberian Husky (*n* = 1). Weight ranged from 8 to 59 kg, with a median of 30.0 kg (interquartile range [IQR]: 23.0-35.2). Age ranged from 5 to 15 years, with a median of 9.0 years (IQR: 7–11.5).

Group C included eight mongrels and 23 other dogs from 15 defined breeds, i.e., Beagle (*n* = 5), Brittany (*n* = 2), German Shepherd (*n* = 2), Golden Retriever (*n* = 2), Cocker Spaniel (*n* = 1), Drever (*n* = 1), Estrela Mountain Dog (*n* = 1), French Bulldog (*n* = 1), German Spitz Klein (*n* = 1), German Spitz Mittel (*n* = 1), Labrador Retriever (*n* = 1), Miniature Pinscher (*n* = 1), Portuguese Pointer (*n* = 1), Portuguese Water Dog (*n* = 1), St. Bernard (*n* = 1) and Transmontano Mastiff (*n* = 1). Weight ranged from 4 to 70 kg, with a median of 19.0 kg (IQR: 13.0-33.0). Age ranged from 1 to 12 years, with a median of 5.0 years (IQR: 2.0-7.0).

### Canine prostate-specific arginine esterase (CPSE)

Blood was collected from all the dogs, left to clot for 4 h at room temperature and the collected serum kept frozen at −25 °C until use. In order to measure plasma CPSE concentrations, an enzyme-linked immunosorbent assay (ELISA) was used strictly following the manufacturer’s instructions (Odelis® CPSE, Virbac, France). Briefly, dilutions of the ELISA stock solution were done to obtain the different calibrator solutions (20, 10, 5.0, 2.5 and 0.0 ng/ml) and to define the standard curve. All the plasma samples were diluted at 1:10 and incubated at 37 °C for 1 h before being assayed. The horseradish peroxidase conjugate were placed into each well after washing cycle and incubated again at 37 °C for 1 h. After another washing cycle, tetramethylbenzidine was added for 10 min and the reaction then stopped. The optical density (“OD”) of each sample was read at 450 nm and CPSE plasma concentrations calculated based on the standard curve plotted with calibrator concentrations. A CPSE value of 61 ng/ml was set as the cut-off for BPH (i.e., values equal to or greater that the cut-off were regarded as positive), according to the manufacturer’s instructions.

### Data analysis

The agreement beyond chance between binomial results (i.e., positive or negative) of CPSE and those of the clinical, ultrasonographic and cytological evaluations for BPH was measured with Cohen’s kappa coefficient (*k*). Sensitivity (true positive [TP]/TP + false negative), specificity (true negative [TN]/TN + false positive), positive likelihood ratio (sensitivity/1 – specificity) and negative likelihood ratio (1 – sensitivity/specificity) were calculated for CPSE binomial results taking cytology as the reference method. Exact binomial 95% confidence intervals (CI) were established for proportions. Differences in the CPSE levels between groups A and C were assessed by means of the Mann-Whitney *U* test. The association between CPSE levels and other quantitative variables (i.e., age, PV and EV) was measured by Spearman’s rank correlation coefficient *r* [17]. Analyses were performed with SPSS 21.0 software for Windows, with a *p* value < 0.05 as statistically significant.

## Results

### CPSE levels between groups

**Fig. 3:**
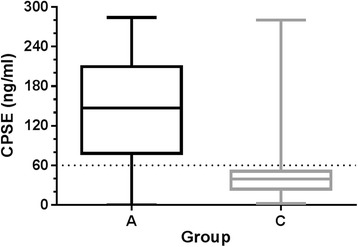
Canine prostate-specific arginine esterase (CPSE) levels in 29 dogs (group A) with benign prostatic hyperplasia (BPH), as diagnosed by cytology; and in 31 dogs (group C) without BPH (*p* < 0.001). The *dotted line* represents the cut-off value for CPSE (i.e., values ≥ 61 ng/ml were regarded as a positive result)

The levels of CPSE (Fig. 3) were significantly different (*p* < 0.001) between group A (median: 160.7 ng/ml; IQR: 80.8–220.5) and group C (median: 29.1 ng/ml; IQR: 19.3–45.5). Significant (*p* < 0.001) correlations were found between age and CPSE level (*r* = 0.549) and CPSE and PV (*r* = 0.448); but not between CPSE and EV (*r* = 0.193; *p* = 0.139).

### Clinical, ultrasonographic and cytological records

In Group A (study group), all 29 dogs evidenced BPH confirmed by cytology; moreover, eight (of these) dogs showed prostatitis concomitant with BPH (proved by the presence of a large number of neutrophils and associated macrophages, and variable amounts of uniform clusters of prostatic cells with middle anysokaryosis and increased size, round and small nucleus, with a clear or moderately basophilic cytoplasm, a characteristic of prostatitis with BPH). In Group C (control group; 31 dogs without BPH proved by cytology), six dogs had prostatitis and two other had squamous metaplasia (presence of large epithelial prostatic cells with a clear to basophilic cytoplasm, and a small dense nucleus similar to that of keratinocytes).

### Comparison between CPSE levels, clinical, ultrasonographic and cytological evaluations

The *k* values from comparisons between binomial CPSE levels and clinic evaluation, ultrasonography and cytology were 0.83, 0.83 and 0.87, respectively (Table 3). A *k* value above 0.80 represents an almost perfect agreement beyond chance.

**Table 3:** Comparison between the binomial results of canine prostate-specific arginine esterase (CPSE; a value ≥ 61 ng/ml was regarded as positive) and those of clinical evaluation, ultrasonography and cytology for the diagnosis of benign prostatic hyperplasia (BPH) in dogs

CPSE^a, b, c^	Clinical evaluation^a^		Ultrasonography^b^		Cytology^c^	
	+ve	–ve	+ve	–ve	+ve	–ve
+ve	30	1	29	2	28	3
–ve	4	25	3	26	1	28
Total	34	26	32	28	29	31

^a^: *k* = 0.83; ^b^: *k* = 0.83; ^c^: *k* = 0.87; +ve: positive; −ve: negative

Three out of the 31 dogs of group C had a positive CPSE result. Two of those three dogs had prostatitis and CPSE values of 174.0 and 183.0 ng/ml; the other animal had squamous metaplasia and a CPSE value of 147.0 ng/ml.

One dog of group A had a CPSE level of 45.2 ng/ml. Sensitivity of the CPSE binomial results (positive or negative) was 96.6% (95% CI: 82.2–99.4), specificity 90.3% (95% CI: 74.2–97.9), positive likelihood ratio 10.0 (95% CI: 3.4–29.3) and negative likelihood ratio 0.04 (95% CI: 0.01–0.26). A positive likelihood ratio of 10.0 reveals a moderate to large and often conclusive increase in the likelihood of BPH if the CPSE result is positive. On the other side, a negative likelihood ratio of 0.04 stands for a large and often conclusive decrease in the likelihood of BPH if the CPSE result is negative.

## Discussion

Prostatic diseases are frequent age-related conditions in intact dogs. The importance of applying a systematic diagnostic protocol that allows an accurate identification of prostatic disorders should be emphasized, as this provides the chance of an early control of the disorder without development of chronic disease, which is hard to manage and causes deterioration of fertility [18].

Several diagnostic methods can be used to evaluate the prostate, from physical examination (i.e., DRE) to ultrasonography, assessment of ejaculate’s third fraction, prostatic massage, prostatic FNA and biopsy [19]. Data obtained from clinical examination are important as starting points for further evaluation of the prostate. Nevertheless, clinical signs of prostatic disease can be transitory and unspecific, precluding a desirably early and conclusive diagnosis. Assessment of the ejaculate’s third fraction could have been an adequate criterion to evaluate prostatic disease. However, taking into account that males included in the study were not breed dogs, semen collection was not attempted. Prostatic ultrasonography is an excellent method in the evaluation of the position, size, contours, and parenchymal echogenicity, as well as in the evaluation of the prostatic urethra. Jointly, clinical evaluation of the prostate and ultrasonography could allow a presumptive diagnosis of the prostatic condition in a reasonable number of cases, but sensitivity would not be optimal (i.e., the ability of detecting all the positive cases). Other methods should then be carried out, such as the prostatic massage and/or the invasive FNA and biopsy [15]. Ultrasound-guided transabdominal FNA was used in this study as the preferred method to obtain prostatic tissue and fluid, and then as the gold standard to which the other methods were compared, considering the very high agreement between FNA and histopathological diagnosis [20] and also the availability of this technique. Besides, the thin monolayer obtained with cytological smears often allows better assessment of individual cytomorphology, as well as improved detection of etiologic agents [21]. Prostatic biopsy is only considered in cases where less invasive diagnostic tests do not render a conclusive diagnosis [6, 19].

Under the above-mentioned circumstances, when it comes to obtain an accurate diagnosis prior to initiating a treatment, several tests can be carried out, taking always into account the benefits, costs and risks of each procedure. Also considering the fact that there is still room for improvement, novel molecular tools have been proposed to early detect prostatic diseases such as the CPSE ELISA kit, which allows determination of CPSE levels in an expeditious manner. In the present study, CPSE levels were determined after the formation of two defined groups, i.e., a study and a control, in which animals were cytologically confirmed as having BPH or not having-BPH, respectively. CPSE serum values were significantly higher in BPH than in non-BPH dogs, which indicate an increased production and secretion of this marker by prostatic epithelial cells in animals with BPH, and further confirm the diagnostic value of the test. Furthermore, seven dogs in group A (BPH positive) had at least one clinical sign but no changes at DRE, while seven other showed no clinical signs. Considering that these dogs were older than 5 years old, the present study highlights the importance of systematic CPSE quantification in middle-aged dogs even when apparently healthy. An early BPH diagnosis in these dogs is always desirable considering the impact of the disorder in fertility [22]. CPSE determination is furthermore useful when ultrasonography is not available or for diagnosis in large and obese dogs in which DRE could be impossible, and also of great utility in the follow-up of BPH medical treatment. CPSE determination does not preclude the attainment of other complementary tests used to exclude the coexistence of other prostatic diseases, such as prostatitis, squamous metaplasia or neoplasia, which are especially recommended when clinical examination and/or ultrasonography are suggestive of disorders other than BPH.

CPSE levels were moderately correlated with prostatic volume (PV), which could be explained by the elevated secretion of CPSE in enlarged hyperplasic prostates. There was no correlation between CPSE and the expected volume (EV) of the prostate, as the latter adjusts prostate size to the weight of dogs. Another important finding is the fact that, although there were significant differences between median weights of the dogs in the two groups, no statistically significant correlation was detected between weight and CPSE levels, a fact which indicates reliability of the test for large breeds.

In group A (study), seven out of 29 dogs with BPH – i.e., with high CPSE values – also presented prostatitis. The association of various prostatic disorders is not uncommon; in fact, diseased prostate glands are commonly affected by multiple and even co-existing pathological conditions [18]. CPH could predispose prostate to inflammation by providing a favorable medium for bacterial growth or by interference with normal defense mechanisms [4]. The hypothesis that inflammation emerges as the major contributor for the development of BPH has been suggested in men [23]. On the other hand, in group C (control), six out of 31 dogs presented prostatitis, with two of them showing CPSE increased values, which could indicate the need of following up these animals. Previous studies have found no difference between serum concentrations of CPSE in dogs with BPH and in those with bacterial prostatitis and carcinoma, which was attributed to the existence of concurrent BPH [11]. However, the method for CPSE determination was different from the current one. In the present study, this could be excluded considering that cytological analysis ruled out BPH in group C. The remaining dogs of group C with prostatitis showed low CPSE values, like the dogs without BPH in the same group. Further studies with large numbers of dogs should contribute to clarify these findings.

## Conclusions

Results of the present study revealed a very high agreement (i.e., almost perfect) between CPSE test and other diagnostic tools for BPH diagnosis. Previous studies have suggested cytological evaluation as one of the methods with higher accuracy in the diagnosis of prostatic diseases [20], which was also observed in the present study, with the highest agreement found between the CPSE test and cytological diagnosis. The CPSE test can very accurately differentiate between dogs with and without BPH and may be considered as an alternative or complementary tool to conventional methods for BPH diagnosis. Finally, its systematic use can increase the likelihood of early diagnosing BPH in apparently healthy middle-aged dogs.

## Additional files

Additional file 1: (“Group A_Hyperplasic dogs”): signalment, clinical diagnosis, ultrasonographic features, cytological diagnosis, prostatic dimensions and CPSE levels in 29 dogs with BPH as determined by cytology. (PDF 37 kb)

Additional file 2: (“Group C_Control Dogs”): signalment, clinical diagnosis, ultrasonographic features, cytological diagnosis, prostatic dimensions and CPSE levels in 31 dogs without evidence of BPH by cytology. (PDF 46 kb)

## Abbreviations

BPH: Benign prostatic hyperplasia
CI: Confidence interval
CPH: Cystic prostatic hyperplasia
CPSE: Canine prostate-specific arginine esterase
ELISA: Enzyme-linked immunosorbent assay
EV: Expected volume
FNA: Fine needle-aspiration
IQR: Interquartile range
PV: Prostatic volume
RDE: Rectal digital examination
