# Supplementary material for: Evaluation of biomarker canine-prostate specific arginine esterase (CPSE) for the diagnosis of benign prostatic hyperplasia
Source: BMC Vet Res. 2017 Mar 23;13:76. doi: 10.1186/s12917-017-0996-5 (PMC5364560; doi:10.1186/s12917-017-0996-5)
Supplement: Supplementary file 1 — (“Group A_Hyperplasic dogs”): signalment, clinical diagnosis, ultrasonographic features, cytological diagnosis, prostatic dimensions and CPSE levels in 29 dogs with BPH as determined by cytology. (PDF 37 kb) [file 12917_2017_996_MOESM1_ESM.pdf]

Group A

				Clinical diagnosis		Ultrasonographic features			Cytological diagnosis						Prostatic dimentions		CPSE(ng/mL)
**Dog**	Breed	Weight	Age	Clinical signs	Rectal Touch	BPH	CBHP	Prostatitis	BPH	Prostatitis	C. prostatitis	Metaplasia	Adenocarcinoma	Normal	Exp. V.	Real V.	
1	Mongrel	23	11	0	1	0	1	0	1	0	0	0	0	0	13.72	84.24	**256**
2	Estrela Mountain Dog	40	9	0	1	0	1	0	1	0	0	0	0	0	18	40.39	**126**
3	Boxer	29.4	11	1	1	0	1	0	1	0	0	0	0	0	15.48	44.53	**284.19**
4	Dalmatian	31.45	8	0	1	0	1	0	1	1	0	0	0	0	15.97	66.30	**230**
5	Brittany	17.5	7	1	1	0	1	0	1	1	0	0	0	0	12.65	20.00	**260**
6	Portuguese Sheepdog	24.2	6	1	0	0	1	0	1	0	1	0	0	0	14.24	54.26	**225**
7	Boxer	34.3	8	1	0	0	1	0	1	0	0	0	0	0	16.64	18.25	**91**
8	Estrela Mountain Dog	43.6	5	1	0	0	1	0	1	0	0	0	0	0	18.86	165.45	**216**
9	L. Retriever	30	7	1	1	0	1	0	1	0	0	0	0	0	16.57	39.23	**69.26**
10	Mongrel	25	7	1	0	0	1	0	1	0	0	0	0	0	14.43	56.07	**203**
11	Estrela Mountain Dog	59	8	1	0	0	1	0	1	1	0	0	0	0	22.28	129.23	**264**
12	Cocker Spaniel	12.3	6	1	1	0	1	0	1	0	0	0	0	0	11.41	15.50	**79**
13	Mongrel	25	14	1	1	0	1	0	1	1	0	0	0	0	14.43	45.78	**76.8**
14	Pekingese	7.8	12	1	1	0	1	0	1	0	0	0	0	0	10.34	19.07	**200.4**
15	Rottweiller	45	6	1	1	1	0	0	1	0	0	0	0	0	19.19	119.60	**203.4**
16	Mongrel	35	11	1	1	0	1	0	1	0	0	0	0	0	16.81	55.56	**103.3**
17	L. Retriever	33	13	1	1	0	1	0	1	0	0	0	0	0	16.33	101.31	**45.2**
18	L. Retriever	35.4	9	0	1	1	0	0	1	0	0	0	0	0	16.91	76.13	**182.3**
19	Siberian Husky	32	9	1	1	1	0	0	1	0	0	0	0	0	16.10	27.99	**82.58**
20	Chow-Chow	26.5	13	1	1	1	0	0	1	0	0	0	0	0	14.79	10.61	**138.5**
21	L. Retriever	35	8	1	0	1	0	0	1	0	0	0	0	0	16.81	13.86	**70.4**
22	Boxer	30	9	1	1	1	0	0	1	0	0	0	0	0	15.62	19.41	**179.45**
23	Golden Retriever	43.1	12	0	1	1	0	0	1	0	0	0	0	0	18.74	45.42	**124.37**
24	Golden Retriever	36.6	12	1	1	0	1	0	1	1	0	0	0	0	17.19	17.08	**68.62**
25	Mongrel	28	10	1	1	0	1	0	1	0	1	0	0	0	15.14	33.22	**138.7**
26	Mongrel	10	15	0	1	1	0	0	1	0	0	0	0	0	10.86	24.92	**186.2**
27	Mongrel	20	10	1	1	1	0	0	1	0	0	0	0	0	13.24	39.58	**160.7**
28	Mongrel	12.3	6	1	1	0	1	0	1	0	0	0	0	0	11.41	16.46	**279.7**
29	Beagle	10.55	5	0	0	0	0	0	1	1	0	0	0	0	11.24	19.96	**64**

BPH Benign prostatic hyperplasia
CBHP Cystic benign prostatic hyperplasia
C. prostatitis Chronic prostatitis
Exp. V. Expected prostatic volume
Real V. Real prostatic volume
CPSE Canine prostate-specific arginine esterase
