# Supplementary material for: Evaluation of biomarker canine-prostate specific arginine esterase (CPSE) for the diagnosis of benign prostatic hyperplasia
Source: BMC Vet Res. 2017 Mar 23;13:76. doi: 10.1186/s12917-017-0996-5 (PMC5364560; doi:10.1186/s12917-017-0996-5)
Supplement: Supplementary file 2 — (“Group C_Control Dogs”): signalment, clinical diagnosis, ultrasonographic features, cytological diagnosis, prostatic dimensions and CPSE levels in 31 dogs without evidence of BPH by cytology. (PDF 46 kb) [file 12917_2017_996_MOESM2_ESM.pdf]

				Clinical diagnosis		Ultrasonographic features			Cytological diagnosis						Prostatic dimentions		CPSE(ng/mL)
**Group C**																	
**Dog**	Breed	Weight	Age (y)	Clinical signs	Rectal Touch	BPH	CBHP	Prostatitis	BPH	Prostatitis	C. prostatitis	Metaplasia	Adenocarcinoma	Normal	Exp. V.	Real V.	
1	German Sheperd	39	3	0	0	0	0	0	0	0	0	0	0	1	17.76	27.80	**41**
2	Beagle	19	1	0	0	0	0	0	0	0	0	0	0	1	13.00	9.15	**6**
3	Drever	17.5	1	0	0	0	0	0	0	0	0	0	0	1	12.65	15.21	**29**
4	Beagle	11.53	1	0	0	0	0	0	0	0	0	0	0	1	11.22	20.57	**45**
5	Golden Retriever	35.5	5	0	0	0	0	0	0	0	0	0	0	1	16.93	18.85	**42.2**
6	St. Bernard	70.2	8	0	0	0	0	0	0	0	0	0	0	1	25.19	79.45	**38.9**
7	Brittany	14.5	5	0	0	0	0	0	0	0	0	0	0	1	11.93	25.81	**44.5**
8	Miniature Pinscher	6	6	0	0	0	0	0	0	0	0	0	0	1	9.91	2.24	**27.93**
9	Portuguese Pointer	22	6	0	0	0	0	0	0	0	0	0	0	1	13.72	19.20	**31.87**
10	Cocker Spaniel	13	6	0	0	0	0	0	0	0	0	0	0	1	11.57	16.18	**23.6**
11	Golden Retriever	33	7	0	0	0	0	0	0	0	0	0	0	1	16.33	31.14	**21.1**
12	Mongrel	14.8	2.5	0	0	0	0	0	0	0	0	0	0	1	12.00	25.82	**29.1**
13	French Bulldog	12	2	0	0	0	0	0	0	0	0	0	0	1	11.34	8.70	**1.8**
14	German Spitz Mittel	10	2	0	0	0	0	0	0	0	0	0	0	1	10.87	11.67	**2.3**
15	Mongrel	15	2	0	0	0	0	0	0	0	0	0	0	1	12.05	26.10	**3.5**
16	Mongrel	31	3	0	0	0	0	0	0	0	0	0	0	1	15.86	40.00	**27.93**
17	Mongrel	21.5	3	0	0	0	0	0	0	0	0	0	0	1	13.60	27.46	**28.3**
18	Brittany	15.5	3	0	0	0	0	0	0	0	0	0	0	1	12.17	25.94	**59.1**
19	Portuguese Water Dog	24	2	0	0	0	0	0	0	0	0	0	0	1	14.19	23.37	**25.4**
20	German Spitz Klein	3.8	2	0	0	0	0	0	0	0	0	0	0	1	9.38	8.80	**47.2**
21	Beagle	20	1	0	0	0	0	0	0	1	0	0	0	0	13.24	12.87	**14**
22	Beagle	19	1	0	0	0	0	0	0	1	0	0	0	0	13.00	8.72	**8**
23	Mongrel	26.2	6	0	0	0	0	0	0	0	0	0	0	1	14.72	18.53	**51**
24	L. Retriever	35	6	0	0	0	0	0	0	0	0	0	0	1	16.81	29.21	**51**
25	Estrela Mountain Dog	61.2	7	0	0	0	0	0	0	0	0	0	0	1	23.05	34	**46.9**
26	German Shepherd	34	11	1	1	1	0	0	0	0	1	0	0	0	16.57	12.60	**21.1**
27	Mongrel	6	6	1	0	0	0	0	0	0	0	0	0	0	9.91	10.77	**46.3**
28	Beagle	14.2	12	0	1	0	1	0	0	1	0	0	0	0	11.86	33.74	**183**
29	Mongrel	7.7	10	1	1	0	1	0	0	0	0	1	0	0	10.31	14.95	**147**
30	Transmontano Mastiff	68	7	1	0	0	0	1	0	1	0	0	0	0	24.66	73.04	**174**
31	Mongrel	20.1	12	1	1	0	1	0	0	1	0	1	0	0	13.26	146.67	**0**

BPH Benign prostatic hyperplasia
CBHP Cystic benign prostatic hyperplasia
C. prostatitis Chronic prostatitis
Exp. V. Expected prostatic volume
Real V. Real prostatic volume
CPSE Canine prostate-specific arginine esterase
